# Supplementary material for: How young children integrate information sources to infer the meaning of words
Source: Nat Hum Behav. 2021 Jul 1;5(8):1046–54. doi: 10.1038/s41562-021-01145-1 (PMC8373611; doi:10.1038/s41562-021-01145-1)
Supplement: Supplementary file 1 — Supplementary Figs. 1–12, Supplementary Tables 1–6 and Supplementary Analysis. [file 41562_2021_1145_MOESM1_ESM.pdf]

nature portfolio

https://doi.org/10.1038/s41562-021-01145-1

Supplementary information

How young children integrate information sources to infer the meaning of words

In the format provided by the authors and unedited

nature portfolio

https://doi.org/10.1038/s41562-021-01145-1

Supplementary information

How young children integrate information sources to infer the meaning of words

In the format provided by the authors and unedited

How young children integrate information sources to infer the meaning of words

Supplementary material

Manuel Bohn, Michael Henry Tessler, Megan Merrick and Michael C. Frank

Contents

Overview **1**

Empirical studies **2**

Experiment 1: Mutual exclusivity 2

Experiment 2: Common ground 5

Experiment 3: Combination 6

Discussion 7

Cognitive models **8**

Modeling framework 8

Loci of development 9

Implementation details 10

Prediction 12

Explanation 15

Summary **18**

Appendix: Model parameters **20**

Semantic knowledge 20

Speaker informativeness and sensitivity to common ground 20

Overview

The goal of this study is to investigate the integration of information during word learning in children between 2 and 5 years of age. As a first step, we replicate earlier work showing that children rely on different forms of pragmatic and semantic information during word learning. In Experiment 1, we show that children make a mutual exclusivity inference and that this inference depends on children's developing semantic knowledge. In Experiment 2, we show that children make inferences about word meanings based on common ground. In Experiment 3, we show that, when combined in one procedure, children are sensitive to the way that the two inferences are aligned.

Next, we introduce a computational cognitive model (**rational integration model**) in which we formalize the process of how information sources are integrated. As part of this, we identify three information sources that children consider when making the alleged inferences: semantic knowledge, expectations about speaker informativeness and sensitivity to common ground. We then use the modeling framework to ask which of these information sources are necessary to predict children's responses in Experiment 3.

In the final section, we turn to the process by which information is integrated. We contrast the process of Bayesian inference we introduced in our model with a biased integration process in which some information sources are weighted as more important. As part of this, we also explore alternative ways to think about developmental change in the integration process.

Empirical studies

Experiment 1: Mutual exclusivity

The first experiment tested the mutual exclusivity inference in children between 2 and 5 years of age. The general inference can be described as follows: when presented with a familiar and an unfamiliar object, children expect a novel word to refer to the unfamiliar object (e.g. Markman and Wachtel 1988). A range of explanations have been put forward for the cognitive basis of this inference (see Lewis et al. 2020 for a discussion). Here, we treat mutual exclusivity as a pragmatic phenomenon (e.g. Clark 1987). The inference process is specified in the model below.

The first goal of this experiment was to quantify developmental change in the age range tested. The second goal was to test the role of semantic knowledge (cf. Lewis et al. 2020). The assumption is that the strength of the mutual exclusivity inference varies with knowledge of the word for the familiar object presented alongside the novel object. That is, when the familiar object is an object for which children are less likely to know the word, they are less likely to assume that the novel word refers to the unfamiliar object. To test this, we systematically varied the familiar object that were presented alongside the novel object.

The experiment was preregistered at https://osf.io/gy37b. The experiment itself can be run by downloading the associated repository (https://github.com/manuelbohn/spin) and opening the file `experiments/ex1_me.html`.

Participants

We tested a total number of 90 children, including 30 2-year-olds (range = 2.03 - 3.00, 15 girls), 30 3-year-olds (range = 3.03 - 3.97, 22 girls) and 30 4-year-olds (range = 4.03 - 4.90, 16 girls). Data from 10 additional children was not included because they were either exposed to less than 75% of English at home (5), did not finish at least half of the test trials (2), the technical equipment failed (2) or their parents reported an autism spectrum disorder (1). All children were recruited from the floor of a Children's museum in San José, California, USA. This population is characterized by diverse ethnic background (predominantly White, Asian, or mixed ethnicity) and high levels of parental education and socioeconomic status. Parents consented to their children's participation and provided demographic information. All experiments were approved by the Stanford Institutional Review Board (protocol no. 19960).

Procedure

The experiment was presented as an interactive picture book on a tablet computer (Frank et al. 2016). Figure 1A shows the general setup. Children saw an animal standing on a little hill between two tables. For each animal character, we recorded a set of utterances (one native English speaker per animal) that were used to talk to the child and make requests. Each experiment started with two training trials in which the speaker requested known objects (car and ball).

In Experiment 1, on one table, there was a familiar object, on the other table, there was a novel object (drawn for the purpose of the study). The speaker requested an object by saying "Oh cool, there is a [non-word] on the table, how neat, can you give me the [non-word]?" Children responded by touching one of the objects. The location of the novel object (left or right table) and the animal character were counterbalanced. We coded as correct choice if children chose the novel object as the referent of the novel word.

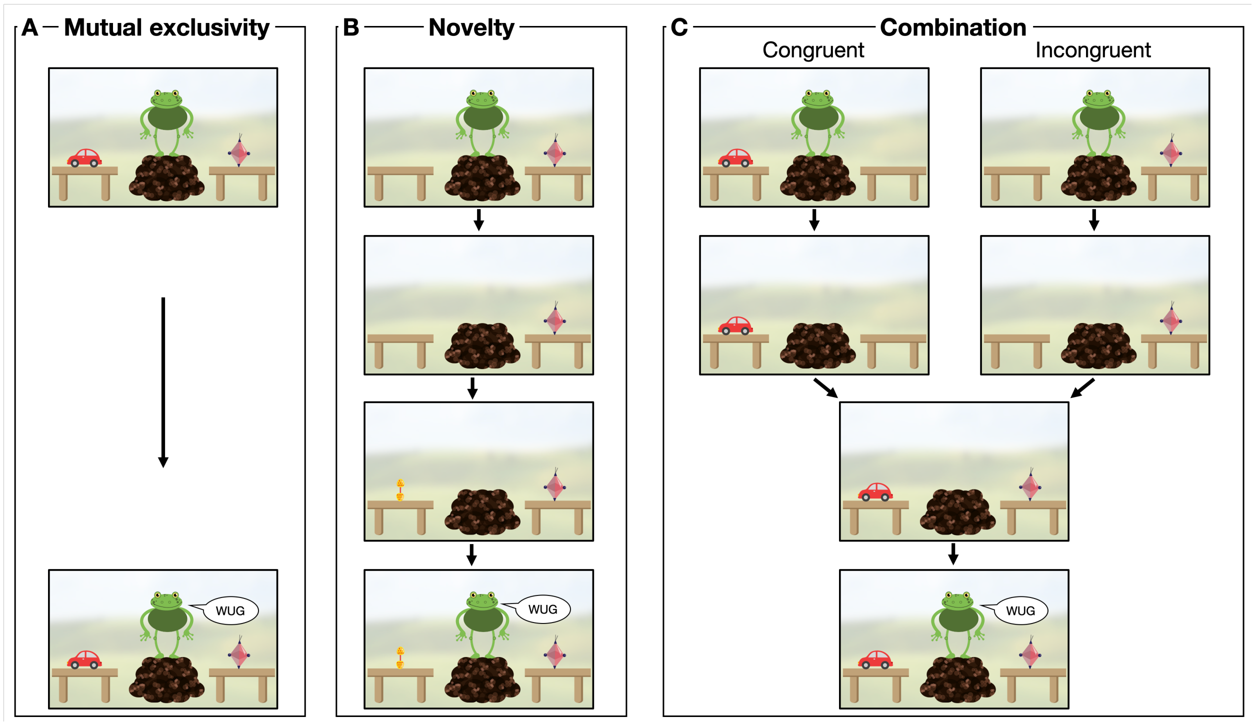

Supplementary Figure 1: Schematic experimental procedure with screenshots from the experiments.

Each child completed 12 trials, each with a different familiar and a different novel object. Familiar objects were selected to vary along the dimension of how likely children were to know the word for each object. This including objects that most 2-year-olds can name (e.g. a duck) as well as objects that only very few 5-year-olds can name (e.g. a pawn). The selection was based on age of acquisition ratings from Kuperman and colleagues (2012). While these ratings do not capture the absolute age when children acquire these words, they capture the relative order in which words are learned. Figure 2A shows the words and objects used in the experiment. We induced this variation to estimate the role of semantic knowledge in a mutual exclusivity inference.

Results

As a first step, we evaluated whether children made a mutual exclusivity inference. For this analysis, we aggregated participants' responses across familiar objects. We used the function `ttestBF` from the R-package `BayesFactor` (Morey and Rouder 2018) to compute a Bayes Factor (BF) in favor of the hypothesis that children chose the novel object more often than expected by chance (50%). Table 1 shows that all age groups made the inference.

As a second step, we investigated how the inference changed as a function of age and the familiar object. We modeled the trial by trial data using a Bayesian generalized linear mixed model (GLMM). We used the function `brm` from the package `brms` (Bürkner 2017). We pre-registered the use of default priors in all models. However, the model in Experiment 3 was unable to initialize with default priors and we thus used weakly informative priors for all models to be consistent. The priors we used were $\mathcal{N}(0,5)$ for fixed (population level) effects and Cauchy$(0,1)$ for standard deviations of random effects. The model formula

Supplementary Table 1: Proportion of children choosing the novel object compared to a level expected by chance based on a one sample Bayesian t-test. Responses are aggregated for each participant across familiar objects.

Age group	Mean	BayesFactor
2	0.61	132
3	0.73	185881356
4	0.86	72514087738

Supplementary Table 2: Model comparison in Experiment 1 based on WAIC scores and weights.

Model	WAIC	SE	weight
with object as RE	1089.05	32.19	1.00
without object as RE	1202.46	31.28	0.00

was `correct ~ age + (1 | id) + (age | object) + (age | agent)`. That is, we modeled an overall slope for age (continuous, anchored at the minimum) and the object specific developmental trajectories as deviations from the overall intercept and slope (random effects). We did not pre-register agent as a random effect, but retrospectively included it to be consistent with Experiment 2 and 3.

The estimate for age was positive and reliably different from zero (β = 0.91, 95% CrI: 0.58 - 1.3). Older children were more likely to make a mutual exclusivity inference. To assess the variability across objects, we compared the fit of the above model to a model lacking `object` as a random effect. Following McElreath (2016), we compared models using WAIC (widely applicable information criterion) scores and weights. The WAIC score is an indicator of the model's predictive accuracy for out of sample data; model's with lower scores are preferred. WAIC weights are an estimate of the probability that this model (compared to all other models considered) will make the best predictions on new data. The model including object provided a much better fit compared to the model lacking it (see Table 2). Figure 2B visualizes the model based developmental trajectory for each familiar object and illustrates the substantial variation between them, both in terms of absolute strength of the inference as well as its developmental trajectory. Figure 2C shows the correlation between rated age of acquisition and object specific model intercept (i.e. mutual exclusivity inference at age 2.00). The mutual exclusivity effect was stronger for words that were rated to be acquired earlier. Objects for which children were less likely to know the word produced a weaker mutual exclusivity effect. Taken together, the strength of the mutual exclusivity inference depended on age as well as the familiar object.

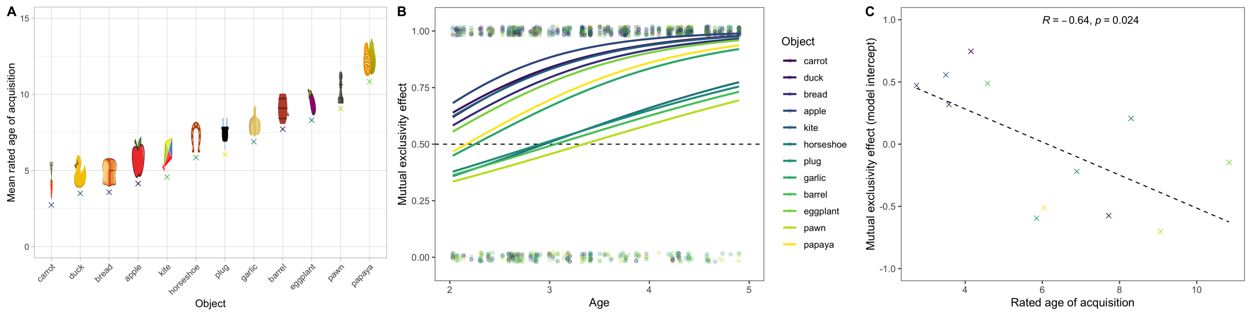

Supplementary Figure 2: A:Familiar words and corresponding pictures by rated age of acquisition. B: Developmental trajectories of mututal exclusivity effect by familiar object based on the mean of the model posterior distribution. Dots show individual datapoints. Lighter colors indicate later rated age of acquisition. Dotted line indicates a level of performance expected by chance. C: Correlation between rated age of acquisiton and mutual exclusivity effect (model based intercept for each familiar object).

Supplementary Table 3: Proportion of children choosing the object that was new to the speaker compared to a level expected by chance based on a one sample Bayesian t-test. Responses are aggregated for each participant across trials.

Age group	Mean	BayesFactor
2	0.55	0.4
3	0.76	26.55
4	0.83	6956.06

Experiment 2: Common ground

Here we tested children's sensitivity to common ground that is build up over the course of a conversation. In particular, we tested whether children keep track of which object is new to a speaker and which they have encountered previously (Akhtar, Carpenter, and Tomasello 1996; Diesendruck et al. 2004). The main goal of the experiment was to measure how children's sensitivity to common ground changes with age.

The experiment was preregistered at https://osf.io/au5hr. The experiment itself can be run by downloading the associated repository and opening the file `experiments/ex2_novel.html`.

Participants

We tested 58 children from the same general population as in Experiment 1, including 18 2-year-olds (range = 2.02 - 2.93, 7 girls), 19 3-year-olds (range = 3.01 - 3.90, 14 girls) and 21 4-year-olds (range = 4.07 - 4.93, 14 girls). Data from 5 additional children was not included because they were either exposed to less than 75% of English at home (3) or the technical equipment failed (2).

Procedure

The general setup was the same as in Experiment 1. The speaker was positioned between the tables. There was a novel object (drawn for the purpose of the study) on one of the tables while the other table was empty. Next, the speaker turned to one of the tables and either commented on the presence ("Aha, look at that.") or the absence ("Hm, nothing there") of an object. Then the speaker disappeared. While the speaker was away, a second novel object appeared on the previously empty table. Then the speaker returned and requested an object in the same way as in Experiment 1 (see also Figure 1B). The positioning of the novel object in the beginning of the experiment, the speaker as well as the location the speaker turned to first was counterbalanced. Children received ten trials, each with a different pair of novel objects. We coded as correct choice if children chose the object that was new to the speaker as the referent of the novel word.

Results

Table 3 compares children's correct responses to a level expected by chance (50%). We found evidence that, as a group, 3- and 4-year-olds, but not 2-year-olds, inferred that the novel word referred to the object that was new to the speaker.

To directly investigate whether children's response changed with age, we modeled the trial by trial data using a Bayesian GLMM (formula: `correct ~ age + (1 | id) + (age | speaker)`, specifications see Experiment 1). The estimate for age was positive and reliably different from zero ($\beta = 0.92$, 95% CrI: 0.37 - 1.54, see Figure 3A). Older children were more likely to chose the object that was new to the speaker as the referent of the novel word, suggesting that the sensitivity to common ground in this context increases with age.

Experiment 3: Combination

Experiment 3 combined the procedures from Experiment 1 and 2. As a consequence, children had to consider not just their semantic knowledge of the word for the familiar object and the inference this licenses but also the role that each object (novel and familiar) had played in the preceding interaction. Combining the two procedures created two alignment conditions: In the *congruent condition*, the novel object was also the object that was new to the speaker. In this case, the mutual exclusivity inference as well as the common ground inference pointed to the novel object as the referent. In the *incongurent condition*, the familiar object was new to the speaker. In this case, the two inferences pointed to different objects. The main focus of the overall study was to model how children integrate and balance these different information sources. We investigate this question in depth in the modelling section below. Here, we limit the discussion to whether children differentiated between the two conditions.

The experiment was preregistered at https://osf.io/4nm8g. The experiment itself can be run by downloading the associated repository and opening the file `experiments/ex3_combination.html`.

Participants

We tested 220 children from the same general population as in Experiment 1 and 2, including 76 2-year-olds (range = 2.04 - 2.99, 7 girls), 72 3-year-olds (range = 3.00 - 3.98, 14 girls) and 72 4-year-olds (range = 4.00 - 4.94, 14 girls). Data from 20 additional children was not included because they were either exposed to less than 75% of English at home (15), did not finish at least half of the test trials (3) or the technical equipment failed (2).

Procedure

Experiment 3 followed the same procedure as Experiment 2 but involved the same objects as Experiment 1 (Figure 1C). In the beginning, one table was empty while there was an object (novel or familiar) on the other one. After commenting on the presence or absence of an object on each table, the speaker disappeared and a second object appeared (familiar or novel). Next, the speaker re-appeared and made the usual request.

In the congruent condition, the familiar object was present in the beginning and the novel object appeared while the speaker was away (Figure 1C - left). In this case, both the mutual exclusivity and the common ground inference pointed to the novel object as the referent. In the incongruent condition, the novel object was present in the beginning and the familiar object appeared later. In this case, the two inferences pointed to different objects (Figure 1C - right).

Participants received up to 12 test trials, six in each condition, each with a different familiar and novel object. Familiar objects were the same as in Experiment 1. The positioning of the objects on the tables, the speaker and the location the speaker first turned to were counterbalanced. Participants could stop the experiment after six trials (three per condition). If a participant stopped after half of the trials, we tested an additional participant to reach a pre-registered number of data points per age group (2-, 3- and 4-year-olds).

Results

All results are reported from the perspective of the mutual exclusivity inference (`correct` in the model formula below). In the incongruent condition, high proportions speak to a mutual exclusivity inference and low proportion to a common ground inference. In the congruent condition, both inferences pointed in the same direction. The focus of this experiment was on information integration and we therefore did not compare the performance to chance.

We modeled the trial by trial data in the following way: `correct ~ age * alignment + (alignment | subid) + (age * alignment | object) + (age * alignment | agent)`. We pre-registered to include item as a fixed effect in Experiment 3. However, we chose to model it as a random effect instead because, as

Supplementary Table 4: Model comparison in Experiment 3 based on WAIC scores and weights.

Model	WAIC	SE	weight
with object as RE	2188.59	46.97	1.00
without object as RE	2390.01	44.28	0.00

explained in Experiment 1, items were chosen based on their rated age of acquisition. That is, we assumed that they are not necessarily different kinds but that they represent different locations on a distribution of required semantic knowledge. For further model specifications see Experiment 1).

The estimate for age was reliably positive (β = 0.81, 95% CrI: 0.4 - 1.24). The incongruent condition had a strong negative impact (β = -1.35, 95% CrI: -2.17 - -0.55), showing that children showed a weaker mutual exclusivity inference when common ground information pointed to the other object as the referent. Thus, children differentiated between the two alignment conditions. The interaction term was weakly - though not entirely - negative, suggesting a shallower slope for age in the incongruent condition (β = -0.2, 95% CrI: -0.66 - 0.27). A model lacking `object` as a random effect provided a much poorer fit, suggesting substantial variation across objects (see Table 4). Figure 3B visualizes the model. Taken together, the results show that children responded to the way the two inferences were aligned with one another.

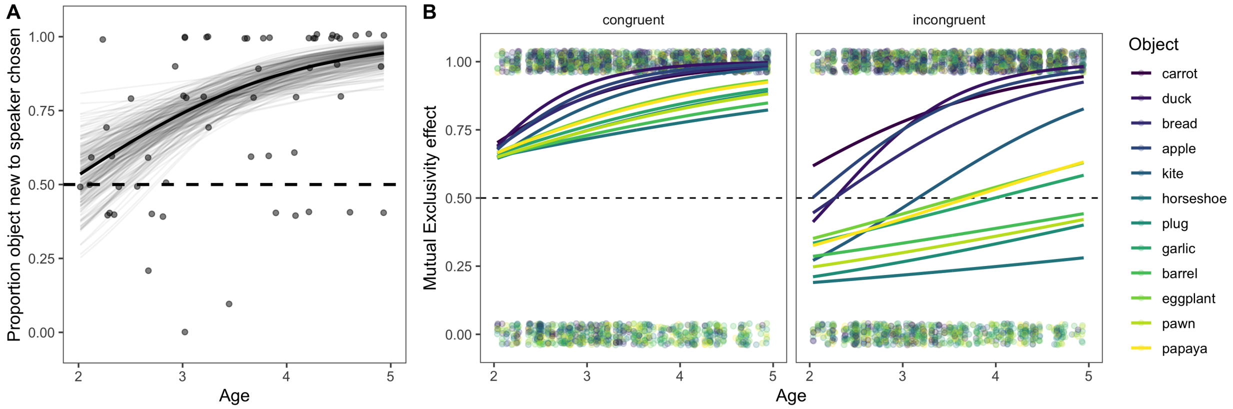

Supplementary Figure 3: Proportion of choosing the object that was new to the speaker by age. Dots show the mean response for each participant. The solid black line shows the developmental trajectory based on the mean of the model posterior distribution. Lighter lines show 200 random draws from the posterior distribution to depict uncertainty. Dotted line indicates a level of performance expected by chance.

Discussion

The experiments reported above show that children are sensitive to the types of information sources we intended to manipulate. Experiment 1 showed that children of all age groups make a mutual exclusivity inference, that the strength of this inference increases with age and, crucially, that it depends on children's semantic knowledge. Experiment 2 showed that children are sensitive to the common ground manipulation we implemented and that the sensitivity to common ground increases with age. Finally, Experiment 3 showed that children respond differently depending on how the mutual exclusivity inference and the common ground inference are aligned with one another. In the next section, we use Bayesian cognitive models to address the question of *how* information sources are integrated when inferences are combined with one another.

Cognitive models

The main purpose of the study was to study *how* children integrate different information sources during word learning and how this process develops with age. To do so, we use Bayesian cognitive models of pragmatic reasoning. We first describe an **rational integration model** which we think best represents the inference and integration processes and then specify how this model captures developmental change. Next, we ask how well this model *predicts* how children integrate information. That is, in a situation in which we know the development trajectories for the mutual exclusivity inference (for a particular familiar object) as well as the common ground inference, what can we say about what happens when they are combined. We then test the predictive power of the model by comparing the model predictions to the data from Experiment 3. We use formal model comparison methods to test the **rational integration model** against a range of alternative models.

Finally, we ask how well our model *explains* the way that children integrate the different information sources. For this analysis, we fit the free parameters in the model to all the available data, those from Experiment 1 and 2 as well as the integration data from Experiment 3. We then compare the model to a range of alternative models that make different assumptions about how information is integrated and how this process develops. This approach answers the question of how we can best explain how children integrate the different information sources.

Modeling framework

The cognitive models are situated in the Rational Speech Act (RSA) framework (Frank and Goodman 2012; Goodman and Frank 2016). RSA models are models of pragmatic reasoning in that they treat language understanding as a special case of Bayesian social reasoning. A listener interprets an utterance by assuming it was produced by a cooperative speaker who had the goal to be informative. Being informative is defined as providing a message that would increase the probability of the listener inferring the speaker's intended message. This notion of contextual informativeness captures the Gricean idea of cooperation between speaker and listener.

$$P_{L_1}(r \mid u) \propto P_{S_1}(u \mid r) \cdot P(r \mid \rho_i) \tag{1}$$

$$P_{S_1}(u \mid r) \propto P_{L_0}(r \mid u)^{\alpha_i} \tag{2}$$

$$P_{L_0}(r \mid u) \propto \mathcal{L}(u, r \mid \theta_{ij}) \tag{3}$$

Our model describes a listener (L_1) reasoning about the referent referred to by a speaker's (S_1) utterance. This reasoning is contextualized by the prior probability of each referent $P(r \mid \rho_i)$. This prior probability is a function of the common ground ρ shared between speaker and listener in that interacting around the objects changes the probability that they will be referred to later. We assume that the degree to which interactions around objects are integrated into the common ground (and thus change the prior probability of those objects) depends on the child's age i.

To decide between referents, the listener (L_1) reasons about what a rational speaker (S_1) would say given an intended referent. This speaker is assumed to compute the informativity for each available utterance and then choose the most informative one. However, this expectation of speaker informativeness may vary and is captured by the parameter α. In particular, we take α to be a function of the child's age i.

The informativity of each utterance is given by imagining which referent a literal listener (L_0), who interprets words according to their lexicon $\mathcal{L}$, would infer upon hearing the utterance.[1] Thus, this reasoning depends

[1]Following Frank and Goodman (2014), we use an implicit uniform prior over referents in the literal listener.

on what kind of semantic knowledge (word–object mappings) the speaker thinks the literal listener knows. We parameterize the listener's knowledge of a word's semantics in terms of a semantic knowledge parameter θ, which varies between 0 and 1. $\theta = 0$ corresponds to the state of knowledge for a completely novel word and results in a semantic interpretation function that chooses randomly between the objects in the scene. Each of the novel words are assumed to have semantic knowledge of 0. For $\theta \in (0, 1)$, the semantic interpretation function will select the familiar referent with probability $\theta + (1 - \theta)\frac{1}{2} = \frac{1+\theta}{2}$; that is, with probability θ, the listener knows the correct meaning of the word (and picks out the correct referent 100% of the time); with probability $1 - \theta$, the listener does not know the meaning of the word and must guess, picking out the correct referent 50% of the time.[2] For familiar objects, semantic knowledge is a function of the degree-of-acquisition of the associated word, which in turn depends upon the word j (its expected acquisition trajectory) as well as on the child's age i.

The model with each data-analytic parameter represented explicitly in {} is:

$$P_{L_1}(r \mid u; \{\rho_i, \alpha_i\, \theta_{ij}\}) \propto P_{S_1}(u \mid r; \{\alpha_i, \theta_{ij}\}) \cdot P(r \mid \rho_i) \tag{4}$$

$$P_{S_1}(u \mid r; \{\alpha_i\, \theta_{ij}\}) \propto P_{L_0}(r \mid u; \{\theta_{ij}\})^{\alpha_i} \tag{5}$$

$$P_{L_0}(r \mid u; \{\theta_{ij}\}) \propto \mathcal{L}(u, r \mid \theta_{ij}) \tag{6}$$

Loci of development

The model description above points to three potential loci of developmental change: semantic knowledge, expectations about speaker informativeness and sensitivity to common ground. Each of theses components is represented by a parameter that plays a particular functional role in the model. We capture developmental change by making these parameters a function of age i for which we estimate a developmental trajectory (intercept and slope in a (logistic-) linear model, see Figure 4).

Semantic knowledge

Semantic knowledge captures the degree of certainty with which the naive listener is assumed to know the label for the familiar object. As a consequence, semantic knowledge differs among familiar words. For objects whose labels are generally acquired earlier (e.g., "carrot") semantic knowledge should generally be high whereas for others (e.g., "pawn") semantic knowledge should generally be lower. However, semantic knowledge also varies with age such that older children are more likely to know the labels for more of the familiar objects compared to younger children. As a consequence, each familiar word has a unique developmental semantic knowledge trajectory.

Technically, the item-specific parameters (θ_{ij}) are estimated in the form of a hierarchical regression (mixed-effects) model: $\theta_{ij} = \text{logistic}(\beta^{\theta}_{0,j} + i \cdot \beta^{\theta}_{1,j})$; each word's lexical development trajectory (the intercept $\beta^{\theta}_{0,j}$ and slope $\beta^{\theta}_{1,j}$ of the regression line for each object) is estimated as a deviation from an overall trajectory of vocabulary development. The intercept and slope for each item are sampled from Gaussian distributions with means $\mu^{\theta}_0, \mu^{\theta}_1$ and variances $\sigma^{\theta}_0, \sigma^{\theta}_1$: $\beta^{\theta}_{0,j} \sim \mathcal{N}(\mu^{\theta}_0, \sigma^{\theta}_0)$ and $\beta^{\theta}_{1,j} \sim \mathcal{N}(\mu^{\theta}_1, \sigma^{\theta}_1)$. μ^{θ}_0 and μ^{θ}_1 represent the overall vocabulary development independent of particular familiar word–object pairings, and σ^{θ}_0 and σ^{θ}_1 represent the overall variability of intercepts and of slopes between items (see Figure 4). For all regression components, intercepts correspond to the knowledge or sensitivity for our youngest children: 2-year-olds (2.00 years of age). For example, μ^{θ}_0 represents the average semantic knowledge of a 2-year-old and μ^{θ}_1

[2]One could also define θ to directly denote the probability of selecting the familiar vs. unfamiliar referent. The model behavior would be the same except for a translation of this parameter. We choose the parametrization described because we find it more intuitive for θ to represent the degree of semantic knowledge, where 0 semantic knowledge represents a state of complete ignorance, corresponding to maximal uncertainty about which object the word applies to.

represents the growth in semantic knowledge over developmental time. σ_0^θ represents the variability in the semantic knowledge of our items (for a 2-year-old) and σ_1^θ represents the variability in growth of semantic knowledge across our different items.

Expectations about speaker informativeness

A second locus of developmental change is a listener's expectations about speaker informativeness α. In the context of the model, speaker informativeness corresponds to the degree with which the listener expects the speaker to choose the most informative of the available utterances. We assume that children at different ages could have different expectations about how rational or informative speakers are (see e.g. Bohn et al. 2019; Frank and Goodman 2014; Yoon and Frank 2019), which we model with a linear function: $\alpha_i = \beta_0^\alpha + i \cdot \beta_1^\alpha$.

Sensitivity to common ground

Sensitivity to common ground ρ refers to the probability that an object is taken to be the referent of the utterance, before actually hearing the utterance. In our paradigm, common ground takes the form of *discourse novelty*, and ρ captures the salience (or lack thereof) of an object due to its presence or absence in the social interaction that precedes the utterance (see 1B). Specifically, the pragmatic listener uses the common ground parameter ρ as the prior probability of the discourse-novel (i.e., the referent that was absent in the social interaction that preceded the utterance): In the *congruent* condition, the object in the prior social interaction (i.e., the discourse-familiar object) was the familiar object, and hence, both common ground (discourse novelty) and mutual exclusivity are cues to the same referent; in the *incongruent* condition, the object in the prior social interaction was the novel object, and hence, common ground (discourse-novelty) and mutual exclusivity are cues to different referents (see Figure 1C).
We expect children at different ages to respond differently to the common ground manipulation, resulting in an age specific prior distribution over objects (Akhtar, Carpenter, and Tomasello 1996; Diesendruck et al. 2004), which we model with a logistic-linear function: $\rho_i = \text{logistic}(\beta_0^\rho + i \cdot \beta_1^\rho)$.

Implementation details

All Bayesian cognitive models were implemented in the probabilistic programming language `WebPPL` (Goodman and Stuhlmüller 2014). The corresponding model code can be found in the associated online repository (file `webppl/prediction_all_models.wppl`). To generate model predictions, we estimated age sensitive parameter distributions for semantic knowledge (by familiar object), speaker informativeness and sensitivity to common ground and then passed them through the model in line with the different ways in which they can be combined and aligned (Figure 4). The resulting predictions come in the form of distributions of developmental trajectories for each familiar object in the congruent and the incongruent condition.

We used the following prior distributions for model parameters. Intercept and slope for sensitivity to common ground: $\beta_0^\rho, \beta_1^\rho \sim \text{Uniform}(-2, 2)$. Speaker informativeness: $\beta_0^\alpha \sim \text{Uniform}(-3, 3)$ for the intercept and $\beta_1^\alpha \sim \text{Uniform}(-0, 4)$ for the slope. We restricted the slope to be positive because negative values for speaker informativeness are conceptually implausible: We do not expect sensitivity to speaker informativeness to decrease across our age range. For the global semantic knowledge (vocabulary) parameters, we used $\mu_0^\theta \sim \text{Uniform}(-3, 3)$ for the intercept and $\mu_1^\theta \sim \text{Uniform}(0, 2)$ for the slope, because it is implausible to assume that semantic knowledge decreases with age. For the parameters capturing the variability the object specific trajectories around these overall parameters we used $\sigma_0^\theta \sim \text{Uniform}(0, 2)$ for the intercept and $\sigma_1^\theta \sim \text{Uniform}(0, 1)$ for the slope. Some choices regarding these prior distributions were made to ease model convergence. However, please note that all models considered used the same prior distributions.

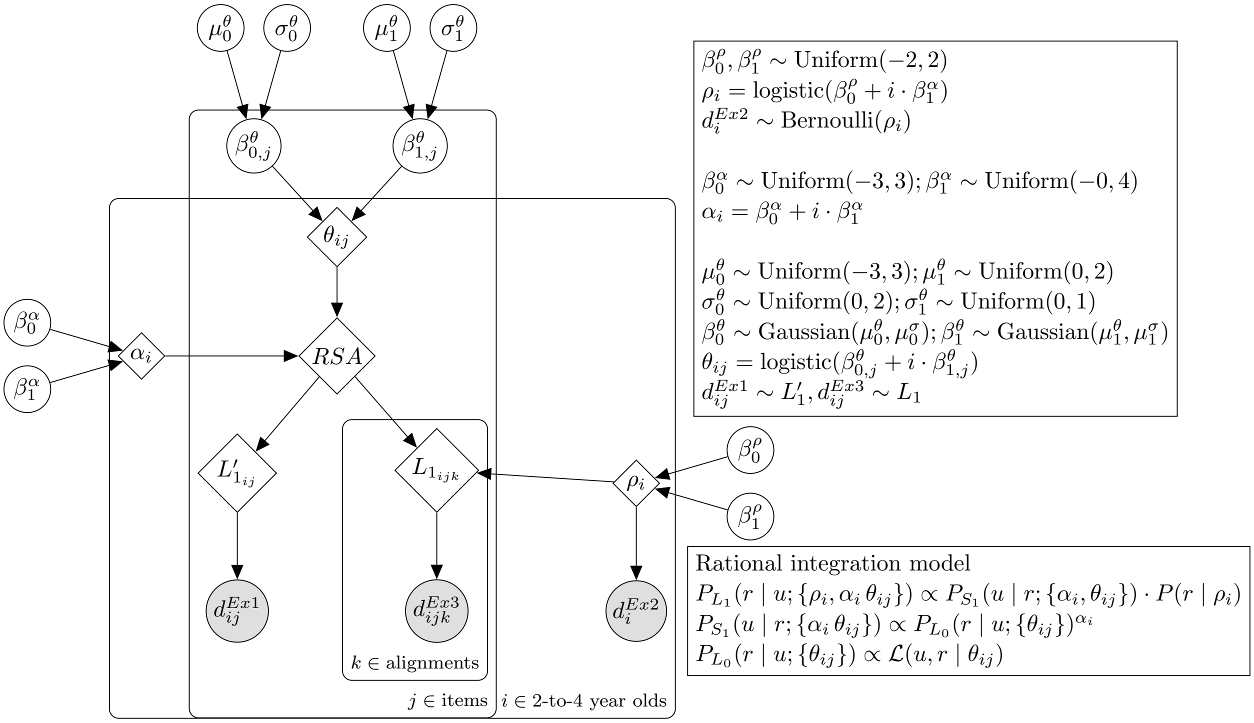

Supplementary Figure 4: Graphical model representing the Bayesian data analysis for **rational integration model** used for *Explanation*. RSA represents the general model architecture and paremeters defined by Eq. (1) - (3). L_1 represents the RSA model defined by Eq. (4), used to predict the combination data d^{Ex3} from Expt. 3 for each unique age bin i, lexical item j, and alignment condition k. This RSA model takes as input a speaker optimality parameter α_i (which varies by age), a semantic knowledge parameter θ_{ij} (which varies by age and item), and a common ground sensitivity ρ_i (which varies by age). Speaker optimality and semantic knowledge parameters are additionally constrained by the data from the Mutual Exclusivity task (d^{Ex1}; Expt. 1), via the same RSA model with no common ground L; common ground sensitivity parameters ρ_i are directly constrained by the data from the Common Ground experiment (d^{Ex2}; Expt. 2). Each of these RSA parameters is sampled from a linear or logistic regression model that track developmental change (with intercepts and slopes given by β_0 and β_1, respectively). Additionally, the semantic knowledge parameters for individual items j are sampled from global vocabulary developmental parameters μ and σ that characterize the mean and standard deviation of the intercepts and slopes for individual item trajectories.

Prediction

In this section we evaluate different models in terms of how well they *predict* information integration. That is, in a situation in which we know the development of the (object specific) mutual exclusivity inference as well as the common ground inference, we look at each model's ability to predict what happens when the two are combined (combination data from Experiment 3). Investigating "pure" (or, *a priori*) prediction automatically excludes all models which include parameters that need to be fit to the combination data itself (e.g., a heuristic, non-integrating biased model, described below). To generate *a priori* predictions, we independently estimated the model parameters for semantic knowledge and speaker informativeness based on Experiment 1 and the parameter for common ground sensitivity based on Experiment 2.

To estimate the parameters for semantic knowledge and speaker informativeness, we adapted the model described above to a situation in which both objects (novel and familiar) have equal prior probability (i.e., no common ground information). We used the data from Experiment 1 to then infer the parameters. That is, we inferred the intercepts and slopes for speaker informativeness (linear regression) and semantic knowledge (logistic regression) that generated RSA model predictions to match the responses generated in Experiment 1. To estimate the parameters representing sensitivity to common ground, we used a simple logistic regression to infer which combination of intercept and slope would generate predictions that corresponded to the average proportion of correct responses measured in Experiment 2. Figure 4 visualizes how parameters were estimated.

To estimate the parameter distributions and generate model predictions, we collected samples from six independent MCMC chains, collecting 150,000 samples from each chain and removing the first 50,000 for burn-in. We excluded samples from one chain because it got stuck on a local maximum which resulted in parameter distributions that were substantially different from the other chains. Model predictions can be downloaded via the following link: https://keeper.mpdl.mpg.de/d/7ae3805dba064bada8e5/. For the model comparison, we collected 150,000 samples (removing 50,000 for burn-in) from ten independent chains (file: `webppl/llh_model.wppl`, see also `stats/model_comparison.Rmd` in the online repository).

Next, we combined the parameters according to the four models described below. Note that the parameter distributions were the same for all models (see Figure 5) and that models only differed in terms of which parameters they included. The models described below are a full model (**rational integration model**) and three lesioned models, which selectively omit one type of information. The following model comparison therefore asks which types of information are necessary to make good predictions about how information is integrated. All models used age-specific parameters when the parameter was present. We do not compare models that make different assumptions about how information is integrated, since they require additional parameters specific to Experiment 3. We consider the question of alternative integration models in the explanation section.

Models

Rational integration model The **rational integration model** serves as the full model and takes in all available information. That is, it takes in object-specific semantic knowledge, speaker informativeness and common ground sensitivity and combines these components by way of the process described above. Figure 6 visualizes the corresponding model predictions in comparison to the data from Experiment 3.

We compare this model to three alternative, "lesioned" models that lack certain components of the rational integration model.

No word-specific knowledge model A marginally less-complex model than the rational integration model uses the same model architecture, taking in speaker informativeness and common ground sensitivity, but omits semantic knowledge that is specific to the familiar objects (i.e., uses only general semantic knowledge). We described above that the parameters for semantic knowledge are fitted via a hierarchical regression (mixed effects) model. In this model, there is an overall developmental trajectory for semantic knowledge (main effect) and then there is object-specific variation around this trajectory (random effects).

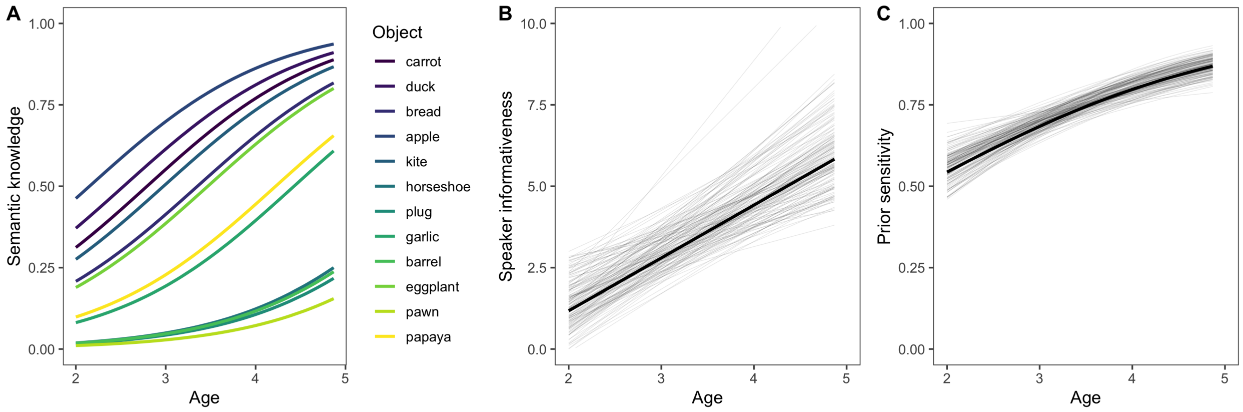

Supplementary Figure 5: Developmental trajectories for model parameters based on the posterior distribution for (A) semantic knowlede, (B) speaker informativeness and (C) prior sensitivity. Solid lines in show the MAP estimate for each parameter. Lighter lines in (B) and (C) show 300 random draws from the posterior distributon to visualize uncertainty. (A) does not include these random draws for the sake of clarity.

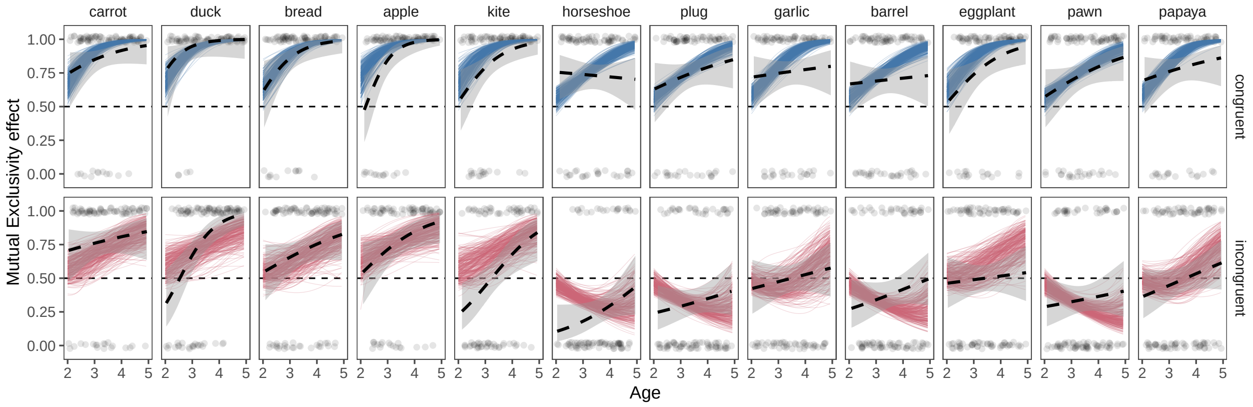

Supplementary Figure 6: Predicting information integration across development. Model predictions based on the rational integration model. Colored lines show developmental trajectories for each familiar object and condition based on 300 random draws from the model posterior distribution. Top row (blue) shows the congruent condition and the bottom row (red) shows the incongruent condition. Familiar objects are ordered based on their rated age of acquisition (left to right). Dashed black lines show smoothed conditional mean of the data with 95% CI (in grey). Light dots are individual data points.

The **no word knowledge model** takes in the overall trajectory for semantic knowledge—represented by μ_0^θ and μ_1^θ—but ignores object-specific variation. Formally, the semantic knowledge for each item is the same: $\theta_i = \mu_0^\theta + i \cdot \mu_1^\theta$. That is, the model assumes a listener whose mutual exclusivity inference does not vary depending on the particular familiar object but only depends on the age specific average semantic knowledge.

$$P_{L_1}^{no\_wk}(r \mid u; \{\rho_i, \alpha_i \, \theta_i\}) \propto P_{S_1}(u \mid r; \{\alpha_i, \theta_i\}) \cdot P(r \mid \rho_i) \tag{7}$$

No common ground model We consider a model that ignores common ground information, ρ_i. This model takes in object specific semantic knowledge and speaker informativeness but uses a prior distribution over objects that is constant across alignment conditions and uniform (e.g., [0.5, 0.5]). This model corresponds to a listener who only focuses on the mutual exclusivity inference and ignores the common ground manipulation. As a consequence, the listener does not differentiate between the two common ground alignment conditions.

$$P_{L_1}^{no\_cg}(r \mid u; \{\alpha_i \, \theta_{ij}\}) \propto P_{S_1}(u \mid r; \{\alpha_i, \theta_{ij}\}) \tag{8}$$

No speaker informativeness model Finally, we consider a model that assumes that the speaker does not communicate in an informative way. This corresponds to $\alpha_i = 0$, which causes the likelihood term to always be 1. As a consequence, this model also ignores semantic knowledge (which affects the likelihood term) and the predictions of this model correspond to the prior distribution over objects.

$$P_{L_1}^{no\_si}(r \mid u; \{\rho_i\}) \propto P(r \mid \rho_i) \tag{9}$$

Model comparison

We compared the models mentioned above in two ways. First, we used correlations between model predictions and the data. For this analysis, we binned the model predictions and the data by age in years and by the type of familiar object. Figure 7 visualizes the correlation between model predictions and the data for all models. The results shows a very high correlation between the predictions of the **rational integration model** and the data in all age groups indicating that the model accurately captures the variation in the data. Correlations for the **rational integration model** were also higher compared to the other models considered. The correlation increased from 2- to 3-year-olds but then again dropped for 4-year-olds. The mismatch for the oldest age group is probably a consequence of the model making very extreme predictions in the congruent condition. This results from the fact that 4-year-olds show very high performance both the mutual exclusivity and common ground tasks. When combined in the model, the two inferences amplify one another because we assume no cost of integration. We maintain no cost to integration because positing integration cost introduces a free parameter that could only be estimated based on the integration data of Experiment 3 itself (i.e., such a model would not make precise *a priori* predictions).

We additionally compared models based on the marginal likelihood of the data under each model – the likelihood of the data averaging over ("marginalizing over") the prior distribution on parameters; the pair-wise ratio of marginal likelihoods for two models is known as the Bayes Factor (see file `model_comparison.Rmd` in the associated online repository). Bayes Factors quantify the quality of predictions of a model, averaging over the possible values of the parameters of the models (weighted by the prior probabilities of those parameter values); by averaging over the prior distribution on parameters, Bayes Factor implicitly take into account model complexity because models with more parameters will tend to have a broader prior distribution over parameters, which in effect, can water down the potential gains in predictive accuracy that a model with more parameters can achieve (Lee and Wagenmakers 2014). For this analysis, we treated age continuously. Table 5 lists the Bayes factors for the different model comparisons. The results show that the **rational integration model**, by far, outperformed all the other models. When comparing the lesioned models among each other,

we see that the more information sources a model takes in, the better predictions it makes (roughly: **no word knowledge** > **no common ground** > **no speaker informativeness**.

Taken together, these analyses showed two things. First, the **rational integration model** makes accurate predictions about how mutual exclusivity and common ground inferences are integrated. It does so based on knowing the strength and development of each inference alone, and incorporating them into a structured probabilistic model of pragmatic reasoning. Second, models that omit one or more types of information (object specific word knowledge, speaker informativeness, common ground sensitivity) make appreciably worse predictions. This result exemplifies that children across the entire age range flexibly integrate all the available information. In the next section we ask investigate alternative formulations of the process of information integration.

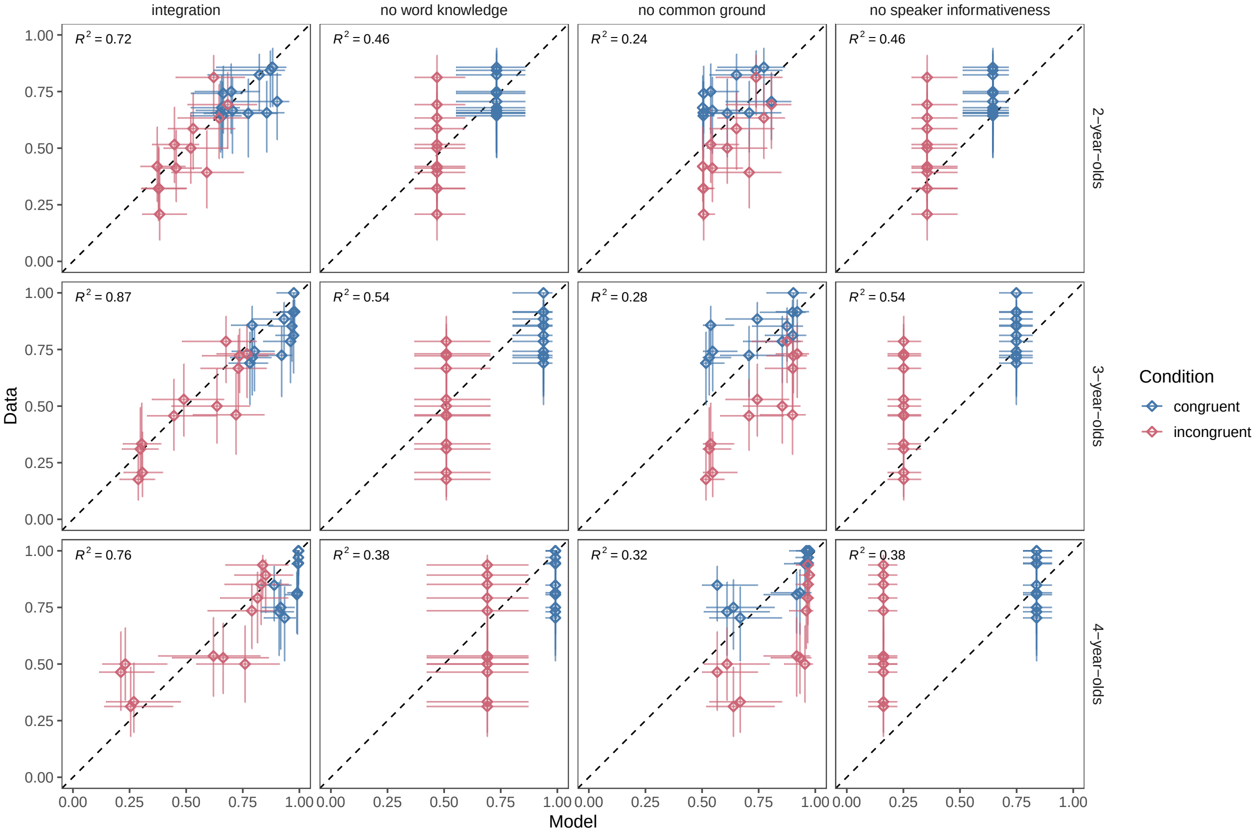

Supplementary Figure 7: Predicting information integration. Correlations between model predictions and data binned by year, item and condition. Vertical and horizontal error bars show 95% HDI. Blue diamonds show congruent condition and red ones show the incongruent condition.

Explanation

In this section we explore how to best explain information integration across development. We explore alternative ways to think about information integration. The **rational integration model** outlined above operates via Bayesian inference in that the prior probability of a referent (a consequence of the common ground manipulation) is updated via the likelihood of hearing the utterance heard from a speaker, which is used to derive the mutual exclusivity inference. In essence, this model is a multiplicative model because the posterior probability of each referent given the utterance is proportional to the product of the likelihood of a speaker saying that utterance and the prior probability of that referent.

Information sources need not be integrated in a Bayesian manner as part of the same pragmatic reasoning

Supplementary Table 5: Model comparison using Bayes factors computed based on the marginal likelihood of each model given the data.

Model comparison	Bayes factor
integration vs no word knowledge	3.924504e+34
integration vs no common ground	2.662902e+46
integration vs no speaker informativeness	4.820386e+109
no word knowledge vs no common ground	6.785321e+11
no word knowledge vs no speaker informativeness	1.228279e+75
no common ground vs no speaker informativeness	1.810201e+63

architecture. Instead, the common ground and mutual exclusivity inferences we consider could be computed separately and combined in a more heuristic fashion. A listener could integrate these two independent inferences in an additive manner, weighting the sources of information by some ratio ϕ. ϕ is then a bias to prefer one type of inference relative to the other. We formalize this alternative hypothesis as a **biased model**.

The **rational integration** and **biased integration** models have potentially different implications for developmental change. The **rational integration model** assumes that the process by which information is integrated remains constant across development. What changes is children's sensitivity to different cues: semantic knowledge, their expectations about speaker informativeness, and their sensitivity to common ground. The **biased integration model** alternatively posits that there is some bias or preference for one information source, and this bias could change across development. This **developmental bias model** makes the same assumptions about developmental change in sensitivities to individual information sources as the **rational integration model** but, in addition, assumes that the way that the mutual exclusivity and the common ground inference are combined changes over time. This model is structurally identical to the **biased model** but the bias parameter ϕ is a function of age, which we model via a logistic-linear model: $\phi_i = \text{logistic}(\beta_0^{\phi} + i \cdot \beta_1^{\phi})$, whose parameters we infer via Bayesian inference.

In this section, we make use of all the available data to arbitrate between these alternative models (a fully Bayesian analysis). As before, Experiments 1 and 2 directly constrain the parameters governing semantic knowledge, speaker informativeness, and the prior distribution over referents (via common ground; Expt. 2). Now, in addition, we incorporate the data from Experiment 3 to additionally constrain these parameters as well as inform the bias parameter for the **biased models** (see Figure 4).

Models

Rational integration model The **rational integration model** in this section differs from the rational integration model in the prediction section only in that the parameter distributions are now additionally informed by the data from Experiment 3. Figure 11 - 12 in the Appendix show how the parameter distributions differ between the prediction and the explanation version of the **rational integration model**. Semantic knowledge and speaker informativeness have similar posterior distributions when taking into account all the data compared to when estimating these parameters only based on Experiment 1 and 2. In contrast, the intercept for common ground sensitivity is estimated to be larger and the slope shallower after taking into account Experiment 3 data. That is, our best guess for common ground sensitivity after taking into account the data from Experiment 3 is that younger children are more sensitive to the common ground manipulation and there is less developmental change. The code to run the model can be found in the associated online repository (file: `webppl/explanation_integration_model.wppl`). Figure 8 shows model predictions for the **rational integration model** in comparison to the data from Experiment 3.

Biased model In the **biased model** the two inferences (common ground and mutual exclusivity) are computed in the same way as in in the **rational integration model**. Subsequently, they are weighted by the bias parameter ϕ:

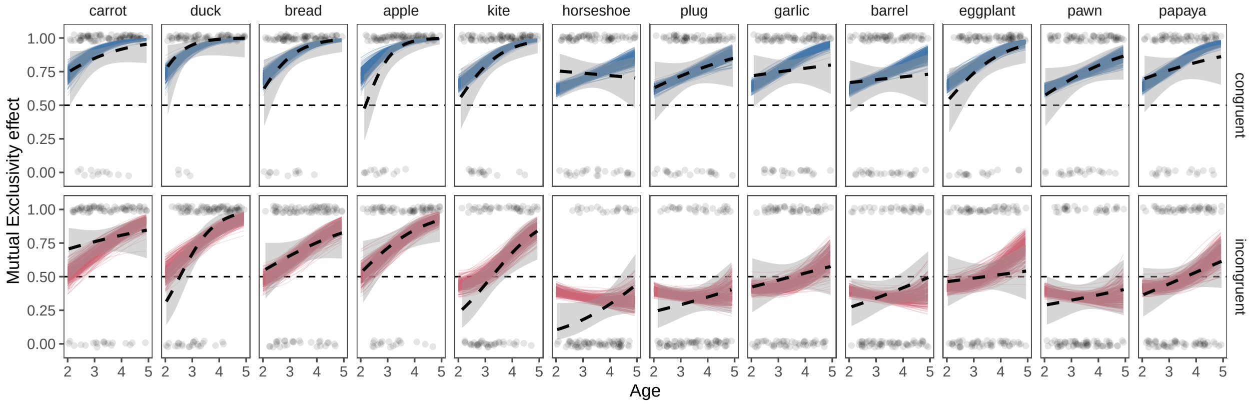

Supplementary Figure 8: Explaining information integration across development. Model predictions based on the rational integration model. Colored lines show developmental trajectories for each familiar object and condition based on 300 random draws from the model posterior distribution. Top row (blue) shows the congruent condition and the bottom row (red) shows the inconguent condition. Familiar objects are ordered based on their rated age of acquisition (left o right). Dashed black lines show smoothed conditional mean of the data with 95% CI (in grey). Light dots are individual data points.

$$P_{L_1}^{biased}(r \mid u; \{\phi, \rho_i, \alpha_i, \theta_{ij}\}) = \phi \cdot P_{ME}(r \mid u; \{\alpha_i, \theta_{ij}\}) + (1 - \phi) \cdot P(r \mid \rho_i) \tag{10}$$

$$P_{ME}(r \mid u; \{\alpha_i, \theta_{ij}\}) \propto P_{S_1}(u \mid r; \{\alpha_i, \theta_{ij}\}) \tag{11}$$

where $P_{ME}(r \mid u; \{\alpha_i, \theta_{ij}\})$ represents a mutual-exclusivity inference from a listener with a uniform prior on referents. $P_{S_1}(u \mid r; \{\alpha_i, \theta_{ij}\}$ is the same as in Eq. (5) above.

To estimate ϕ as well as the other parameters, we make use of all the available data. The model code can be found in the associated online repository (file: `webppl/explanation_bias_model.wppl`). The posterior distribution for the bias parameter ϕ is shown in figure 9A. It suggests that the mutual exclusivity inference is weighted as slightly more important compared to the common ground inference.

Developmental bias model For this model, we make the bias parameter ϕ as a logistic-linear function of age– $\phi_i = \text{logistic}(\beta_0^{\phi} + i \cdot \beta_1^{\phi})$ — and estimate the intercept and slope that yield the best model predictions compared to the data from Experiment 3. The **developmental bias model** is then:

$$P_{L_1}^{dev\_bias}(r \mid u; \{\phi_i, \rho_i, \alpha_i, \theta_{ij}\}) = \phi_i \cdot P_{ME}(r \mid u; \{\alpha_i, \theta_{ij}\}) + (1 - \phi_i) \cdot P(r \mid \rho_i) \tag{12}$$

The model code can be found in the associated online repository (file: `webppl/explanation_dev_bias_model.wppl`). Figure 9B visualizes the developmental trajectory of the bias parameter. Based on this model, the common ground inference seems to decrease in importance compared to the mutual exclusivity inference with age.

Model comparison

As before, we compared models based on correlations and Bayes factors. Figure 10 shows correlations between model predictions and the data, each binned by year and by object. Even though both biased models have more free parameters then the **rational integration model**, the **rational integration model** shows the highest correlation in all age groups, though model predictions and data are closely aligned for all models. Next we directly compared models based using Bayes factors (see Table 6). We also included the prediction **rational integration model** into this analysis.

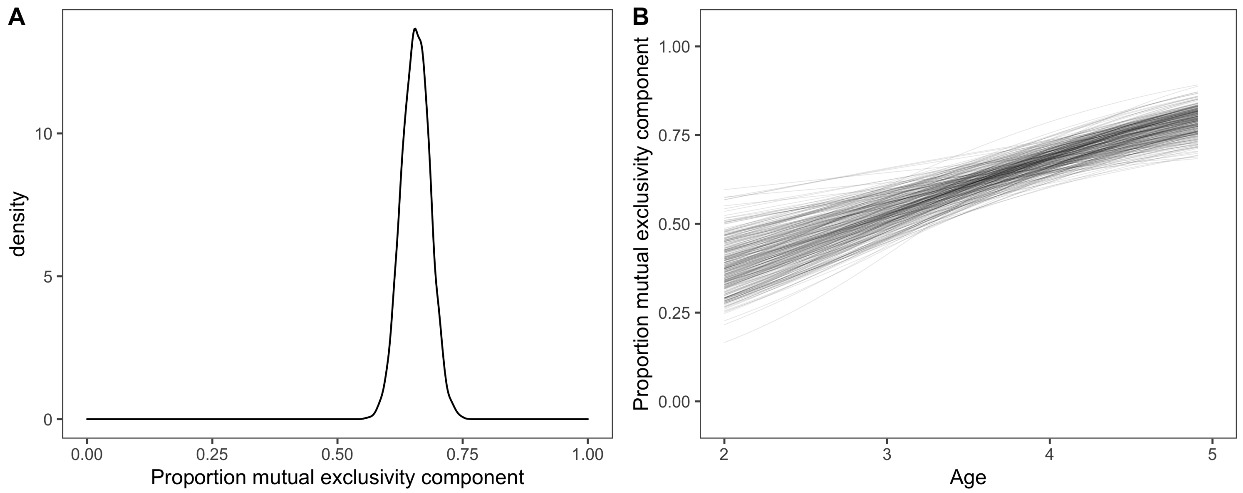

Supplementary Figure 9: Bias parameter for the biased model (A) and the developmental bias model (B). (A) shows the posterior distribution of the bias parameter and (B) shows developmental trajectories for the bias parameter based on 300 random draws from the posterior distribution for intercept and slope.

Supplementary Table 6: Model comparison using Bayes factors computed based on the marginal likelihood of each model given the data.

Model comparison	Bayes factor
integration vs biased	207871411
integration vs developmental bias	1372480
developmental bias vs biased	151

When comparing models via their marginal likelihood, we again see that the **rational integration model** provides, by far, the best fit to the data compared to the two **biased models**, even though the rational integration model has fewer free parameters than the **biased models**. When comparing the two **biased models** directly, we see that an age sensitive bias parameter did not result in a substantially better fit, providing additional evidence that the integration process itself is not changing across development.

This analysis shows that the inference and integration processes described by the **rational integration model** accurately capture the data and also explain information integration better compared to the additive **biased models**. As a consequence, we may say that instead of being biased towards one type of inference, children are rationally integrating all the information sources available.

Summary

Here we studied how 2 to 5 year old children integrate semantic and pragmatic information during word learning. In three experiments, we first showed that children make a mutual exclusivity inference and that this inference varied depending on children's familiarity with the objects involved (Experiment 1). Next, we showed that children make common ground inferences based on their interactions with a speaker (Experiment 2). When the two inferences were combined, we found that children were sensitive to the way in which they were aligned (Experiment 3).

We then introduced a computational model to investigate the process by which children integrated the inferences in Experiment 3. As a start, we described mutual exclusivity as a pragmatic inference, which takes in children's emerging semantic knowledge and their expectations about how informative a speaker is. The **rational integration model** assumes that this inference is then flexibly integrated with children's developing sensitivity to common ground.

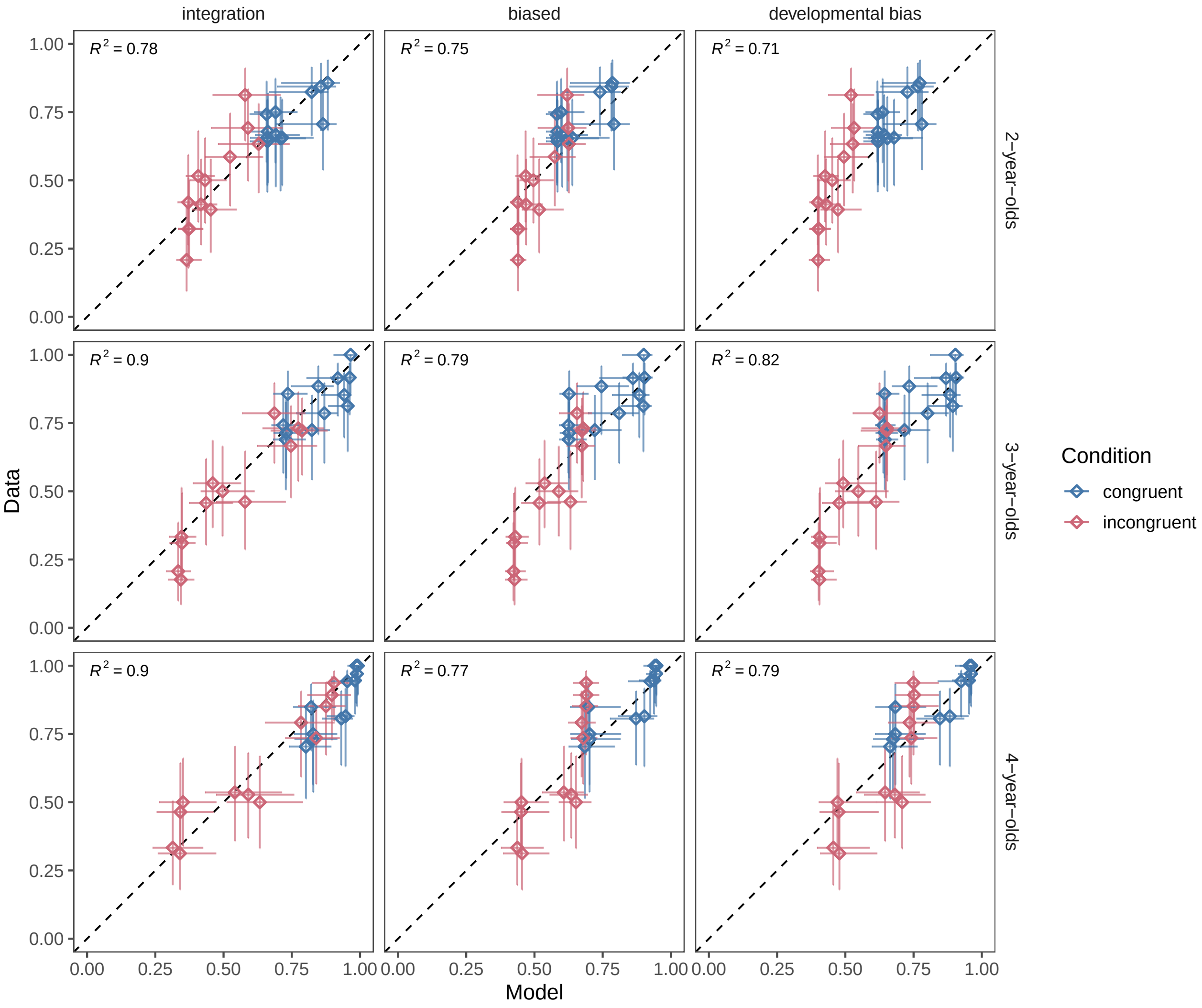

Supplementary Figure 10: Explaining information integration. Correlations between model predictions and data binned by year, item and condition. Vertical and horizontal error bars show 95% HDI. Blue diamonds show congruent condition and red ones show the incongruent condition.

Next, we tested the predictive power of this model. That is, we asked how well the model would predict the data of Experiment 3, when only knowing the developmental trajectories for mutual exclusivity (based on Experiment 1) and common ground (Experiment 2). We found a very close alignment of the model predictions and the data across the entire age range. Furthermore, the **rational integration model** provided a better fit to the data compared to a number of lesioned models, which selectively omitted one type of information. These results suggest that children flexibly integrate all available information.

In the final section, we studied which process best explained children's information integration. We compared the **rational integration model** to a **biased model** which assumed that children are biased towards one type of inference, and a **developmental bias model** that assumed that the bias could change across development. We found that the **rational integration model** better explained the data compared to both of these biased models, even though the biased models had more free parameters. In sum, we found that children's integration of semantic and pragmatic information during word learning is best described as a form of Bayesian social inference.

Appendix: Model parameters

In the following, we visualize the model parameters for semantic knowledge, speaker informativeness and common ground sensitivity. Please note that the alternative lesion models presented in the prediction section used the same parameter distributions as the prediction **rational integration model**.

Semantic knowledge

Intercept

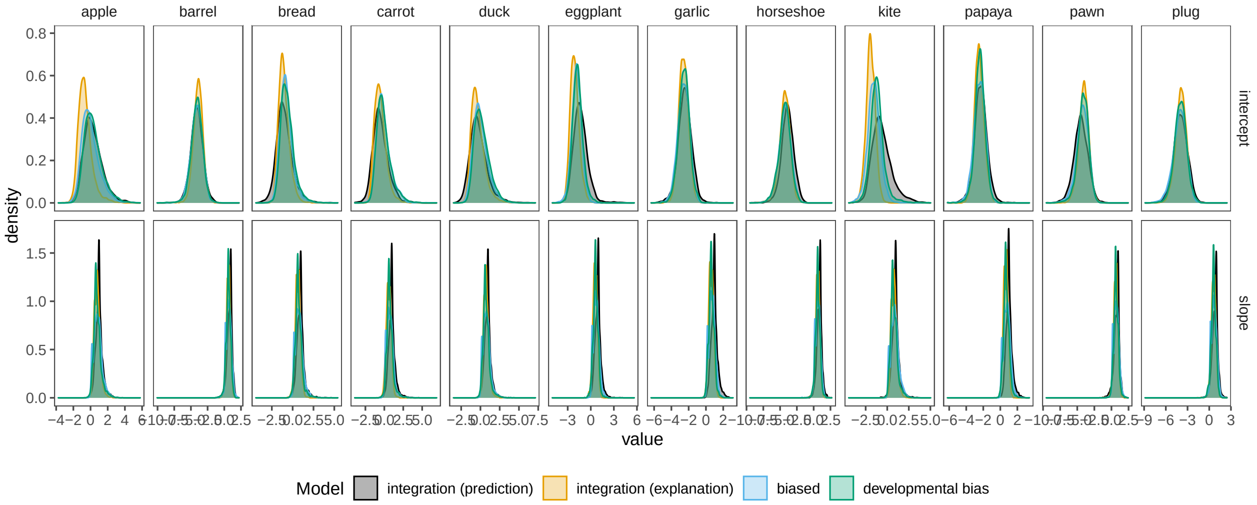

Supplementary Figure 11: Posterior distribution of intercept and slope term for semantic knowledge for each object by model.

Speaker informativeness and sensitivity to common ground

Akhtar, Nameera, Malinda Carpenter, and Michael Tomasello. 1996. "The Role of Discourse Novelty in Early Word Learning." *Child Development* 67 (2): 635–45.

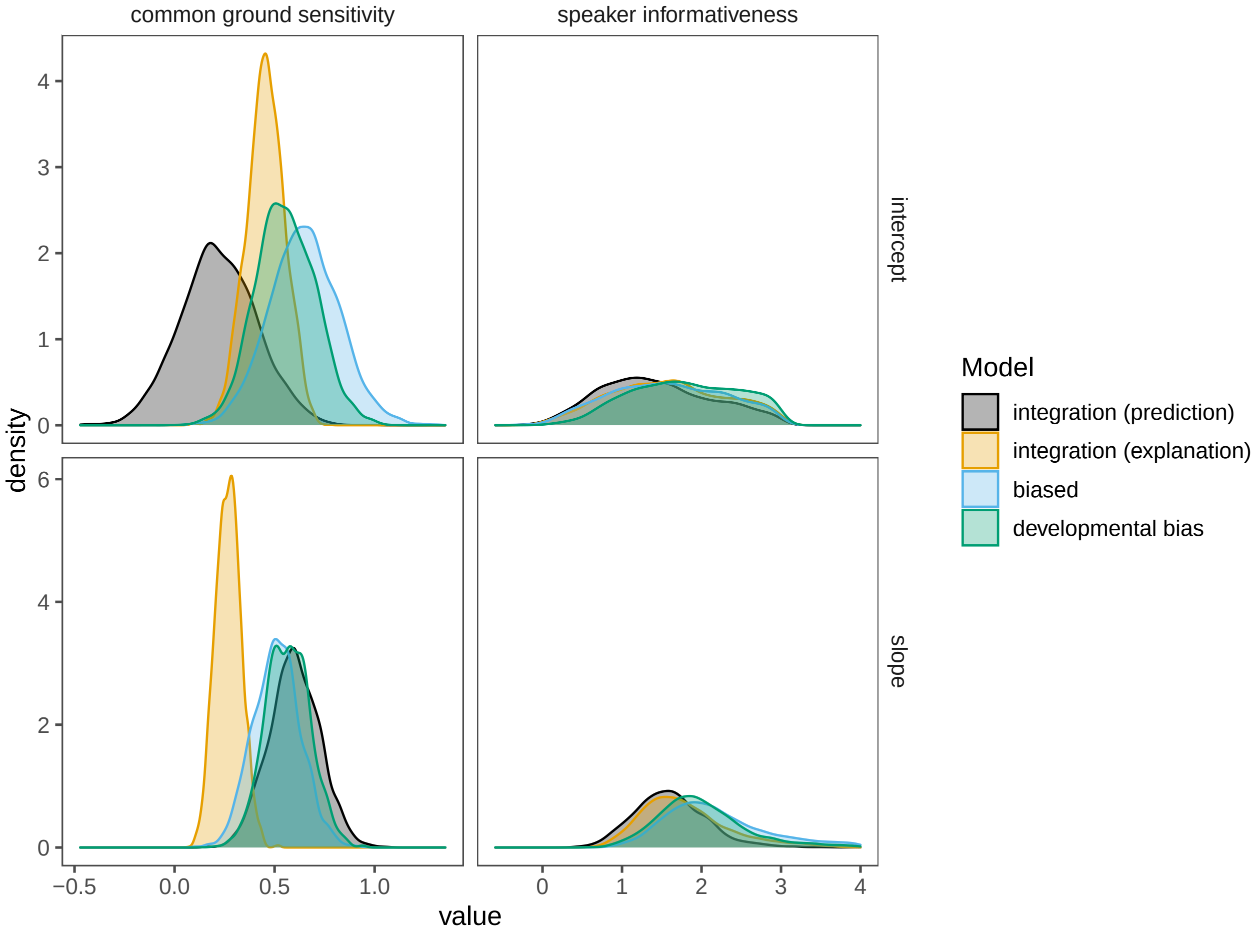

Supplementary Figure 12: Posterior distribution of slope and intercept terms for speaker informativeness and sensitivity to common ground by model.

Bohn, Manuel, Michael H Tessler, Megan Merrick, and Michael C Frank. 2019. "Predicting Pragmatic Cue Integration in Adults' and Children's Inferences about Novel Word Meanings," September. https://doi.org/10.31234/osf.io/xma4f.

Bürkner, Paul-Christian. 2017. "brms: An R Package for Bayesian Multilevel Models Using Stan." *Journal of Statistical Software* 80 (1): 1–28. https://doi.org/10.18637/jss.v080.i01.

Clark, Eve V. 1987. "The Principle of Contrast: A Constraint on Language Acquisition." *Mechanisms of Language Acquisition.*

Diesendruck, Gil, Lori Markson, Nameera Akhtar, and Ayelet Reudor. 2004. "Two-Year-Olds' Sensitivity to Speakers' Intent: An Alternative Account of Samuelson and Smith." *Developmental Science* 7 (1): 33–41.

Frank, Michael C, and Noah D Goodman. 2012. "Predicting Pragmatic Reasoning in Language Games." *Science* 336 (6084): 998–98.

———. 2014. "Inferring Word Meanings by Assuming That Speakers Are Informative." *Cognitive Psychology* 75: 80–96.

Frank, Michael C, Elise Sugarman, Alexandra C Horowitz, Molly L Lewis, and Daniel Yurovsky. 2016. "Using Tablets to Collect Data from Young Children." *Journal of Cognition and Development* 17 (1): 1–17.

Goodman, Noah D, and Michael C Frank. 2016. "Pragmatic Language Interpretation as Probabilistic Inference." *Trends in Cognitive Sciences* 20 (11): 818–29.

Goodman, Noah D, and Andreas Stuhlmüller. 2014. "The design and implementation of probabilistic programming languages." http://dippl.org.

Kuperman, Victor, Hans Stadthagen-Gonzalez, and Marc Brysbaert. 2012. "Age-of-Acquisition Ratings for 30,000 English Words." *Behavior Research Methods* 44 (4): 978–90.

Lee, Michael D, and Eric-Jan Wagenmakers. 2014. *Bayesian Cognitive Modeling: A Practical Course.* Cambridge University Press.

Lewis, Molly L, Veronica Cristiano, Brenden M. Lake, Tammy Kwan, and Michael C Frank. 2020. "The Role of Developmental Change and Linguistic Experience in the Mutual Exclusivity Effect." *Cognition* 198: 104191.

Markman, Ellen M, and Gwyn F Wachtel. 1988. "Children's Use of Mutual Exclusivity to Constrain the Meanings of Words." *Cognitive Psychology* 20 (2): 121–57.

McElreath, Richard. 2016. *Statistical rethinking: A bayesian course with examples in R and Stan.* Texts in Statistical Science. Boca Raton: CRC Press.

Morey, Richard D., and Jeffrey N. Rouder. 2018. *BayesFactor: Computation of Bayes Factors for Common Designs.* https://CRAN.R-project.org/package=BayesFactor.

Yoon, Erica J, and Michael C Frank. 2019. "The Role of Salience in Young Children's Processing of Ad Hoc Implicatures." *Journal of Experimental Child Psychology* 186: 99–116.
