# Supplementary material for: How young children integrate information sources to infer the meaning of words
Source: Nat Hum Behav. 2021 Jul 1;5(8):1046–54. doi: 10.1038/s41562-021-01145-1 (PMC8373611; doi:10.1038/s41562-021-01145-1)
Supplement: Supplementary file 2 — Reporting Summary [file 41562_2021_1145_MOESM2_ESM.pdf]

nature research

Corresponding author(s): Manuel Bohn

Last updated by author(s): May 11, 2021

Reporting Summary

Nature Research wishes to improve the reproducibility of the work that we publish. This form provides structure for consistency and transparency in reporting. For further information on Nature Research policies, see Authors & Referees and the Editorial Policy Checklist.

Statistics

For all statistical analyses, confirm that the following items are present in the figure legend, table legend, main text, or Methods section.

n/a	Confirmed	
☐	☒	The exact sample size (*n*) for each experimental group/condition, given as a discrete number and unit of measurement
☐	☒	A statement on whether measurements were taken from distinct samples or whether the same sample was measured repeatedly
☐	☒	The statistical test(s) used AND whether they are one- or two-sided<br>*Only common tests should be described solely by name; describe more complex techniques in the Methods section.*
☐	☒	A description of all covariates tested
☒	☐	A description of any assumptions or corrections, such as tests of normality and adjustment for multiple comparisons
☐	☒	A full description of the statistical parameters including central tendency (e.g. means) or other basic estimates (e.g. regression coefficient) AND variation (e.g. standard deviation) or associated estimates of uncertainty (e.g. confidence intervals)
☒	☐	For null hypothesis testing, the test statistic (e.g. *F*, *t*, *r*) with confidence intervals, effect sizes, degrees of freedom and *P* value noted<br>*Give P values as exact values whenever suitable.*
☐	☒	For Bayesian analysis, information on the choice of priors and Markov chain Monte Carlo settings
☐	☒	For hierarchical and complex designs, identification of the appropriate level for tests and full reporting of outcomes
☐	☒	Estimates of effect sizes (e.g. Cohen's *d*, Pearson's *r*), indicating how they were calculated

Our web collection on statistics for biologists contains articles on many of the points above.

Software and code

Policy information about availability of computer code

Data collection	All data was collected using custom made picture book style web-experiments (implemented in HTML/ JavaScript). Experiments (and code) can be found in the associated online respository (https://github.com/manuelbohn/spin).
Data analysis	Data processing and model comparisons were done in R. Probabilistic models and model comparisons were implemented in WebPPL. All analysis code can be found in the supplementary material and the associated online repository (https://github.com/manuelbohn/spin).

For manuscripts utilizing custom algorithms or software that are central to the research but not yet described in published literature, software must be made available to editors/reviewers. We strongly encourage code deposition in a community repository (e.g. GitHub). See the Nature Research guidelines for submitting code & software for further information.

Data

Policy information about availability of data

All manuscripts must include a data availability statement. This statement should provide the following information, where applicable:

- Accession codes, unique identifiers, or web links for publicly available datasets
- A list of figures that have associated raw data
- A description of any restrictions on data availability

Experimental stimuli, data files and analysis scripts are freely available in an online repository (https://github.com/manuelbohn/spin)

Field-specific reporting

Please select the one below that is the best fit for your research. If you are not sure, read the appropriate sections before making your selection.

☐ Life sciences ☒ Behavioural & social sciences ☐ Ecological, evolutionary & environmental sciences

October 2018

Behavioural & social sciences study design

All studies must disclose on these points even when the disclosure is negative.

Study description	Quantitative experimental studies with cognitive modelling component
Research sample	Children (N = 368 , age range: 2.0 - 5.0): Visitors to the Children's Discovery Museum in San Jose, California, USA with mixed ethnic background.
Sampling strategy	The sample was a convenience sample. Sample sizes were set based on previous studies and pre-registered prior to data collection.
Data collection	Experiments were presented in the form of a website on a tablet computer. Children were guided through the experiment by an experimenter. The experimenter was blind to the purpose of the study. Two training trials, in which participants had to listen to respond to audio recordings, were placed at the beginning of each experiment to assure that participants paid attention and understood the basic procedure. Parents were present in the room but asked to sit back and fill out a demographics form.
Timing	Data collection for adults took place between March and August 2019.
Data exclusions	Participants were excluded if they responded incorrectly in 2/2 training trials in which they had to select a familiar object (car, ball) upon request. Children were further excluded if they reported to hear less than 75% of English at home or if they finished less than half of the test trials of a given experiment.
Non-participation	Data from 35 additional children were not included because they were either exposed to less than 75% of English at home (23), did not finish at least half of the test trials (5), the technical equipment failed (6) or their parent reported an autism spectrum disorder (1).
Randomization	Critical comparisons were based on conditions tested within subject. The order of conditions within subjects was randomized. Experiments were run in succession and participants were therefore assigned to whichever experiment was running at the moment.

Reporting for specific materials, systems and methods

We require information from authors about some types of materials, experimental systems and methods used in many studies. Here, indicate whether each material, system or method listed is relevant to your study. If you are not sure if a list item applies to your research, read the appropriate section before selecting a response.

Materials & experimental systems

n/a	Involved in the study	
☒	☐	Antibodies
☒	☐	Eukaryotic cell lines
☒	☐	Palaeontology
☒	☐	Animals and other organisms
☐	☒	Human research participants
☒	☐	Clinical data

Methods

n/a	Involved in the study	
☒	☐	ChIP-seq
☒	☐	Flow cytometry
☒	☐	MRI-based neuroimaging

Human research participants

Policy information about studies involving human research participants

Population characteristics	See above
Recruitment	Parents were approached on the museum floor and asked whether they would like to participate in a short experiment on child development. Parents received no monetary compensation for their participation, children received a sticker. Due to its location, visitors to the museum constitute a WEIRD sample with exceptionally high levels of education and SES. The exact age at which children show evidence for making inferences on individual information sources might thus be different in other populations. However, we have no reason to assume that the information integration process, as specified in our model, would be different.
Ethics oversight	Stanford Institutional Review Board, protocol #19960.

Note that full information on the approval of the study protocol must also be provided in the manuscript.
